# Innovation in *Medicine*

**DOI:** 10.3390/medicines1010001

**Published:** 2014-06-10

**Authors:** Gerhard Litscher

**Affiliations:** 1 Research Unit for Complementary and Integrative Laser Medicine, Medical University of Graz, Auenbruggerplatz 29, 8036 Graz, Austria; E-Mail: gerhard.litscher@medunigraz.at; Tel.: +43-316-385-13907; Fax: +43-316-385-13908; 2 Research Unit of Biomedical Engineering in Anesthesia and Intensive Care Medicine, Medical University of Graz, Auenbruggerplatz 29, 8036 Graz, Austria; 3 TCM Research Center Graz, Medical University of Graz, Auenbruggerplatz 29, 8036 Graz, Austria

Medicine and innovation: these terms have always been inseparable. In each phase of medical history, the continuous expansion of medical knowledge and the development of technical solutions went hand in hand. Interdisciplinarity also played an important role from the beginning. Integrative Medicine has become the driving force behind the latest innovations in medicine over recent years.

The journal *Medicines*, launched in 2014, is being published by a Swiss publisher from Basel. Its objective is the publication and wide electronic dissemination of innovative and consequential research in all types of medicine. *Medicines* will publish refereed articles of high quality for a professional audience. The journal thereby aims to promote communication between medical researchers. The Swiss publisher will host the journal’s website, which will be accessible to readers free of charge. The main topics covered include, but are not limited to:

- History of medicine; medicine and philosophy.
- Proven or evidence-based non-conventional (traditional, alternative, complementary or integrative) medicines or ethno-medicines, natural product-based medicines, nutraceuticals.
- The chemistry, medicinal chemistry, structure–activity relationships and computer aided modeling based on naturally occurring agents, either therapeutic or potentially therapeutic.
- Conventional medicine: both established and emerging natural product-based therapeutic agents and treatments.

Therefore, we invite investigators and researchers to submit original research articles as well as review articles associated with medicine. We are particularly interested in articles dealing with eastern/oriental medicine and its possible contributions to western medicine—and *vice versa*.

A very successful example of such a cross-over approach is high-tech acupuncture [1]. Only a few days ago, the 1st World Congress on High-Tech Acupuncture and Integrative Medicine took place in Nanjing, China [2]. Similar to the journal *Medicines*, this annual World Congress [3] shall serve as an opportunity to present and discuss the state of the art of multidisciplinary approaches to modernization of integrative medicine, especially traditional Chinese medicine and acupuncture. The journal and the congress will also be an excellent opportunity to establish research networks and scientific communication on these interesting traditional and innovative medical topics.

Contributors to the journal should be experts and scientists from all over the world. They are welcome to send their most recent research and describe current scientific medical trends.

This is a cordial invitation to all of you to submit high-level articles to our new journal.

## References

[1] Litscher G., Gao X.Y., Wang L., Zhu B. (2012). High-tech acupuncture and Integrative Laser Medicine.

[2] 1st World Congress of High-Tech Acupuncture and Integrative Medicine. http://www.bitlifesciences.com/HTA&IM2014.

[3] 2nd World Congress of High-Tech Acupuncture and Integrative Medicine. http://litscher.info.

